# Frequency and age at occurrence of clinical manifestations of disease in patients with hypophosphatasia: a systematic literature review

**DOI:** 10.1186/s13023-019-1062-0

**Published:** 2019-04-25

**Authors:** Shelagh M. Szabo, Ioannis C. Tomazos, Anna Petryk, Lauren C. Powell, Bonnie M. K. Donato, Yuri A. Zarate, Anatoly Tiulpakov, Gabriel Ángel Martos-Moreno

**Affiliations:** 1 Broadstreet HEOR, Vancouver, BC Canada; 2 Alexion Pharmaceuticals, Inc., Boston, MA USA; 3 Department of Pediatrics, Division of Pediatric Endocrinology, University of Minnesota, Minneapolis, MN USA; 4 Section of Genetics and Metabolism, University of Arkansas for Medical Sciences, Little Rock, AR USA; 5 Endocrinology Research Centre, Moscow, Russia; 6 Department of Endocrinology, Hospital Infantil Universitario Niño Jesús, IIS La Princesa, Madrid, Spain; 7 Department of Pediatrics, Universidad Autónoma de Madrid, Madrid, Spain; 8 CIBERobn, ISCIII, Madrid, Spain

**Keywords:** Burden, Hypophosphatasia, Manifestations, Signs, Symptoms, Systematic literature review

## Abstract

**Background:**

Hypophosphatasia (HPP) is a rare, inherited, metabolic disease caused by tissue-nonspecific alkaline phosphatase deficiency, characterized by bone mineralization defects and systemic complications. Understanding of the clinical course and burden of HPP is limited by its rarity. This systematic literature review and synthesis of case report data aimed to determine the frequency and timing of clinical HPP manifestations and events.

**Methods:**

Case reports and series of patients with HPP who had been followed longitudinally for ≥1 year were identified. Demographics and clinical data of interest, identified through consultation with clinical experts in HPP, were extracted. Occurrences of clinical manifestations/events of interest were categorized, classified by age at first reported occurrence of HPP manifestations and visualized over time. Clinical manifestations/events considered to contribute to the clinical burden of HPP were identified. Kaplan–Meier curves were used to estimate the median (range) age at first occurrence of the most frequently reported manifestations/events.

**Results:**

From the 283 studies that met the inclusion criteria, 265 patients with HPP with ≥1 year of longitudinal follow-up were identified (median [interquartile range] age 4 [0–34] years; 45% male). The types of clinical manifestations/events of interest experienced by individuals with ≥1 such manifestation/event (*n* = 261) often differed between older and younger patients. Most (94%) of the 265 patients experienced ≥1 manifestation/event deemed to contribute to the clinical burden of HPP; premature tooth loss (53.5%), fractures (35.8%), pain (33.6%), and gross motor/ambulation difficulties (30.9%) were most frequently reported. The median (range) age at first reported occurrence of respiratory symptoms, cranial abnormalities, and premature tooth loss ranged from 0.3 to 10 years, whereas the median age at first reported occurrence of fractures, pain, gross motor/ambulation difficulties, and surgery ranged from 33 to 70 years.

**Conclusions:**

HPP is associated with a high clinical burden of disease, regardless of age at first reported occurrence of HPP manifestations. Over an individual’s lifetime, the types of manifestations/events experienced can change and multiple HPP-related clinical manifestations/events can accumulate. These observations may reflect evolution and progression of the disease.

**Electronic supplementary material:**

The online version of this article (10.1186/s13023-019-1062-0) contains supplementary material, which is available to authorized users.

## Background

Hypophosphatasia (HPP) is a rare, systemic, genetic, metabolic disease caused by deficient activity of the tissue non-specific isoenzyme of alkaline phosphatase, resulting in the extracellular accumulation of its substrates [1]. The clinical presentation of HPP can vary considerably between individuals and includes skeletal problems, muscle weakness, ambulatory difficulties, pain, and dental, neurologic, and renal manifestations [2]. Historically, HPP has been classified based on age at first manifestation of the disease, into perinatal, infantile, childhood, and adulthood HPP [3]. HPP identified in patients in the perinatal and infantile periods is associated with significant mortality [4]. Those who survive, and those who present with the disease later, may show manifestations of HPP that include poor growth [5–8], delay in reaching motor milestones [6, 9, 10], muscle weakness [7, 8], pain [11, 12], dental abnormalities [13], and low-trauma fractures [7, 14]. These clinical manifestations often lead to ambulatory difficulties that can restrict activities of daily living, which can contribute to the high burden of disease seen among patients with HPP [7]. In addition, the chronic and heterogeneous nature of the condition means clinical manifestations experienced by individual patients can change over time. For example, some patients who have a history of only odontohypophosphatasia (odonto-HPP) during childhood may develop skeletal manifestations during adulthood [15].

Case reports and case series describing selected outcomes for approximately 500 cases of HPP have been published; however, knowledge of the overall clinical course of HPP is limited. To date, no robust, long-term observational studies assessing the natural history, clinical progression, or outcomes in patients with HPP have been published. Although case reports and case series include data that are not collected in a standardized fashion, they provide a rich source of information about the clinical manifestations patients’ experience at different ages. A synthesis of case report data may increase our understanding of the natural history of the disease.

The aim of this systematic literature review and synthesis of patient case report data was to determine the frequency and timing of key clinical manifestations and events of HPP.

## Methods

### Systematic literature review

A systematic review of the literature was conducted according to best practices, and following Preferred Reporting Items for Systematic Reviews and Meta-Analyses (PRISMA) guidelines [16]. A customized search strategy including the terms ‘hypophosphatasia’ and ‘HPP’ was implemented in PubMed/MEDLINE and Embase (from the inception of the databases in 1950 and 1947, respectively, to February 2017) to identify studies of patients with HPP who had been followed longitudinally for at least 1 year. Eligible study designs included case series and reports in which patient-level data were presented in sufficient detail to understand the age at occurrence and frequency of clinical manifestations, and events of interest (defined below). Animal and biochemical studies were excluded, as well as articles published in languages other than English.

Titles and abstracts of articles were independently screened and categorized as ‘included’, ‘excluded’, or ‘unsure’ by two reviewers; any discrepancies were resolved by a third reviewer who made the final judgment. The full text of articles meeting the inclusion criteria and those categorized as ‘unsure’ were analyzed and the articles were either confirmed to meet the inclusion criteria or excluded. The reference lists of included studies were also screened for relevant articles not captured by the search strategy.

### Data extraction

Data from included articles were extracted into an Excel workbook by two reviewers. If available, demographic characteristics of the patients described were extracted, including: age at diagnosis, family history of HPP, age at loss to follow-up, and age at death (if applicable). Clinical manifestations and events of interest, which were identified through consultation with four clinical experts who had experience in treating patients with HPP, as well as the reported age of their occurrence, were extracted. Author names, year of publication, study design, and the specialty of the treating physician were also included.

The extracted clinical manifestations and events of interest were coded and grouped into the following broad categories after consultation with the clinical experts: skeletal, gross motor/ambulatory difficulties, dental, respiratory, or renal, and are referred to as HPP-related manifestations or events of interest from here onwards (Additional file 1). In addition, clinical manifestations and events of interest that were deemed to contribute to the clinical burden of HPP were also identified after consultation with clinical experts. They were defined as those being sufficiently impactful that they would have likely been described in the case reports had they occurred. These manifestations and events were then grouped, focusing on those that would likely have an important impact on an individual’s activities of daily living, functional status, or emotional status (Table 1).

**Table 1:** HPP-related manifestations/events considered to contribute to the clinical burden of HPP

Manifestation/event	Description
Premature tooth loss	Premature loss of primary and/or permanent teeth
Cranial abnormalities	Craniosynostosis, frontal bossing, large anterior fontanel, decreased skull ossification, increased intracranial pressure, hydrocephalus
Gross motor/ambulation difficulties	Difficulty walking, gait disturbance, need for mobility devices including wheelchair, other mobility impacts, inability to work or impairment of activities of daily living
Surgery	Any HPP-related surgery (including extremity, cranial, dental, spinal, and other [thoracoscopic epiphysiodesis, osteotomy, tracheostomy])
Other dental complications	Delayed eruption of teeth, abnormality of the dentition, severe periodontitis, and atrophy of alveolar ridges
Fractures	Bone fractures, including pseudofracture and stress fracture
Pain	Bone, joint, muscle, and nonspecific/generalized pain
Respiratory symptoms	Respiratory compromise, pulmonary hypoplasia, respiratory failure, recurrent respiratory tract infections, and need for respiratory support
Hospitalizations	Hospitalization for any cause
Kidney complications	Nephrocalcinosis or renal insufficiency
Seizures	Vitamin B6-responsive seizures, seizures of unknown origin and any neurological symptom
Psychological	Depressivity, anxiety, insomnia, or other

HPP-related manifestations and events considered to contribute to the clinical burden of HPP were identified by four clinical experts who had experience in treating patients with HPP

*HPP* hypophosphatasia

### Data analysis

Data on clinical manifestations and events were tabulated for all patients identified in the case reports, overall and according to the age at first reported occurrence of HPP manifestations. The age categories were broadly based on those published by the American Academy of Pediatrics [17], but were also informed by the underlying distribution of the data in order to best observe patterns in disease burden and symptomology: in utero*;* infancy/early childhood (aged less than 2 years; sub-divided to age 0 to less than 0.5 years, and 0.5 to less than 2 years, for selected outcomes); childhood (aged 2 to less than 10 years); adolescence (aged 10 to less than 18 years) and adulthood (aged 18 years or older).

#### Frequency and age at occurrence of all HPP-related clinical manifestations and events over time

Data derived from cases that had both longitudinal follow-up of at least 1 year and had experienced one or more HPP-related manifestations or events of interest were used to visualize HPP-related clinical manifestation/event patterns and the burden of HPP at the individual case level. These data were plotted as grouped clinical manifestations and event categories over time using bar charts; one bar chart was generated for each age group category.

#### Frequency and age at occurrence of HPP-related manifestations and events considered to contribute to the clinical burden of HPP

For cases that had at least 1 year of follow-up available, the frequency of and age at occurrence of manifestations and events that contribute to the clinical burden of HPP were recorded. The number of organ systems (from skeletal, dental, gross motor, renal, and respiratory) affected by those manifestations and events over time was also recorded. Kaplan–Meier (K–M) curves were used to estimate the median (range) age at first occurrence of the following selected HPP manifestations and events: premature tooth loss, fracture (one or more fractures), multiple fractures (three or more fractures), gross motor/ambulation difficulties, pain, surgery, cranial abnormalities, and respiratory symptoms. Censoring was indicated on the K–M curves to account for the fact that follow-up times varied owing to the nature of the case report data, and that the reliability of the estimate of the proportion of patients who did not experience the outcome was dependent on the number of individuals not censored [18]. Death and loss to follow-up were considered to be censoring events. The three most common manifestations and events that were reported in each age group category, regardless of age at first occurrence of HPP manifestation, were included in this analysis.

## Results

### Systematic literature review

The PRISMA flow diagram for the selection of eligible studies is shown in Additional file 2. In total, 3259 abstracts were identified, of which 283 met the study inclusion criteria. From the included studies, 511 individual cases of HPP were identified; longitudinal (at least 1 year) follow-up data were available for 265 of these patients (Additional file 3). A subset of these patients had one or more HPP-related manifestations or events of interest (*n* = 261; Additional files 1 and 3).

### Patient demographics and clinical characteristics

Of the 265 patients with longitudinal follow-up data, 44.5% were male. The median (interquartile range; IQR) age of the patients at the first presentation to the authors of the case reports was 4.0 (0.4–34.0) years (Table 2). The median (IQR) duration of follow-up was 7.0 (3.0–18.0) years. A family history of HPP was reported for 27.9% of patients. The first HPP manifestation occurred most frequently during infancy/early childhood (Table 2).

**Table 2:** Demographic/clinical characteristics and duration of follow-up of patients with ≥1 year of follow-up

Characteristic	Value
Total cases, n (%)	265 (100.0)
Male, n (%)	118 (44.5)
Median (IQR) age at anchor visit, years^a^	4.0 (0.4–34.0)
Reported family history of HPP, n (%)	74 (27.9)
Age at first reported occurrence of HPP manifestations, n (%)	
In utero	30 (11.5)
Infancy/early childhood (< 2 years)	101 (38.7)
Childhood (2 to < 10 years)	78 (29.9)
Adolescence (10 to < 18 years)	9 (3.4)
Adulthood (≥ 18 years)	43 (16.5)
Median (IQR) duration of follow-up by age at first reported occurrence of HPP manifestations, years	
Overall	7.0 (3.0–18.0)
In utero	4.0 (2.1–10.3)
Infancy/early childhood (< 2 years)	5.4 (2.4–14.8)
Childhood (2 to < 10 years)	7.7 (3.6–28.5)
Adolescence (10 to < 18 years)	6.0 (4.3–27.0)
Adulthood (≥ 18 years)	15.0 (6.3–27.0)

All patients who had been followed longitudinally for at least 1 year were included in this analysis

^a^Anchor visit: first presentation to author of the case report

*HPP* hypophosphatasia, *IQR* interquartile range

### Age at occurrence of all HPP-related clinical manifestations and events

**Fig. 1:**
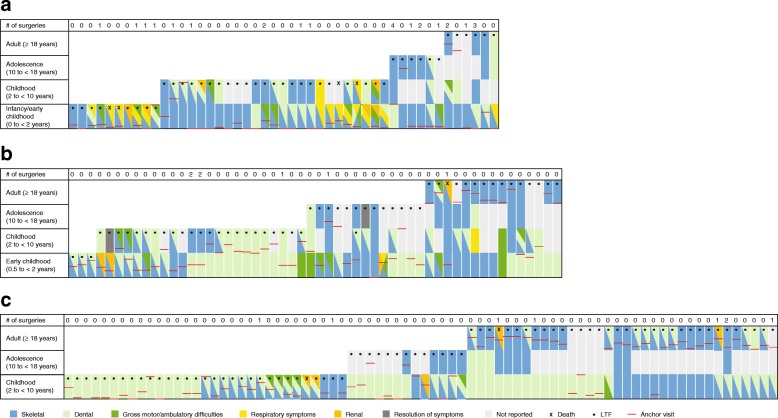
Timing of HPP-related clinical manifestations/events in patients showing first manifestations in (**a**) infancy, (**b**) early childhood, and (**c**) childhood. **a** Infancy; 0 to < 0.5 years (*n* = 47). **b** Early childhood; 0.5 to < 2 years (*n* = 54). **c** Childhood; 2 to < 10 years (*n* = 78). These graphs are examples for patients with HPP in infancy, early childhood, and childhood – see Additional file 4 for additional age groups. Cases are ordered by available follow-up. Only patients who had been followed longitudinally for at least 1 year and who experienced one or more HPP-related manifestations or events of interest were included in this analysis. ‘Resolution of symptoms’ was reported by the author of the case report. ‘Not reported’ defined as either nothing reported for the time period or no manifestations of interest. Anchor visit defined as the first point of contact with the author of the case report. HPP, hypophosphatasia; LTF, lost to follow-up

All HPP-related clinical manifestations and events of interest were mapped over time for individual patients who had both longitudinal follow-up of at least 1 year and had experienced one or more HPP-related manifestations or events of interest (*n* = 261). Evolution of symptomatology for individual patients from these case reports whose first HPP manifestation occurred in infancy, early childhood, and childhood is shown in Fig. 1; similar charts were developed for all age group categories and are included in Additional file 4.

The HPP-related symptoms or events of interest experienced by individual patients often differed between older and younger patients. For example, many patients with first HPP manifestations in childhood initially experienced dental symptoms, while manifestations among patients in adolescence and adulthood were often predominantly skeletal (Fig. 1).

### Frequency of HPP-related manifestations and events considered to contribute to the clinical burden of HPP

Of the 265 patients with at least 1 year of longitudinal follow-up, most (94%) experienced at least one manifestation or event that was considered to contribute to the clinical burden of HPP during follow-up. The most commonly reported clinical manifestations or events across all age groups were (Table 3): premature tooth loss (53.5%), fractures (35.8%), pain (33.6%), and gross motor/ambulation difficulties (30.9%). The three most frequently reported clinical manifestations or events during follow-up for each age at first manifestation group were: in utero (fracture, cranial abnormalities, and gross motor/ambulation difficulties), infancy (premature loss of teeth, cranial abnormalities, and gross motor/ambulation difficulties), childhood (premature loss of teeth, pain, and fracture), adolescence (pain, premature loss of teeth, and fracture) and adulthood (pain, multiple fractures, and gross motor/ambulation difficulties). Cranial abnormalities and respiratory symptoms occurred predominantly among very young patients (in utero and infancy). Hospitalizations occurred across all age groups and ranged from 3% (*n* = 1/30) among patients with HPP manifestations in utero to 22% (*n* = 2/9) among patients with HPP first manifesting in adolescence. Surgical interventions were more frequently reported in adults and adolescents than younger age groups.

**Table 3:** Proportion of patients with manifestations/events considered to contribute to the clinical burden of HPP

	Overall n (%) *n* = 265	In utero n (%) *n* = 30	Infancy n (%) *n* = 102	Childhood n (%) *n* = 78	Adolescence n (%) *n* = 9	Adulthood n (%) *n* = 46
Patients with at least one manifestation/event	248 (94)	25 (83)	96 (94)	78 (100)	9 (100)	40 (87)
Manifestation/event						
Premature loss of teeth	***142 (53.5)***	3 (10.0)	***63 (61.8)***	***58 (74.4)***	***3 (33.3)***	14 (30.4)
One or more fractures	***95 (35.8)***	***12 (40.0)***	19 (18.8)	***27 (34.6)***	***3 (33.3)***	***34 (73.9)***
Multiple fractures^a^	60 (22.6)	2 (6.7)	13 (12.7)	19 (24.4)	2 (22.2)	***24 (52.2)***
Pain	***89 (33.6)***	1 (3.3)	25 (24.5)	***32 (41.0)***	***6 (66.7)***	***25 (54.3)***
Gross motor/ambulation difficulties	82 (30.9)	***7 (23.3)***	***41 (40.2)***	18 (23.1)	1 (11.0)	15 (32.6)
Cranial abnormalities	66 (24.9)	***12 (40.0)***	***44 (43.1)***	10 (12.8)	0 (0.0)	0 (0.0)
Surgery	64 (24.2)	4 (13.3)	30 (29.4)	8 (10.1)	***3 (33.3)***	17 (37.0)
Dental other^b^	37 (14.0)	0 (0.0)	23 (22.5)	11 (14.1)	0 (0.0)	3 (6.5)
Hospitalizations^c^	33 (12.5)	1 (3.3)	18 (17.6)	6 (7.7)	2 (22.2)	6 (13.0)
Respiratory symptoms^c^	19 (7.2)	5 (16.7)	14 (13.7)	0 (0.0)	0 (0.0)	0 (0.0)
Nephrocalcinosis	11 (4.2)	2 (6.7)	8 (7.8)	1 (1.3)	0 (0.0)	0 (0.0)
Seizures	7 (2.6)	3 (10.0)	3 (2.9)	0 (0.0)	0 (0.0)	1 (2.2)
Renal insufficiency	4 (1.5)	0 (0.0)	1 (1.0)	3 (3.8)	0 (0.0)	0 (0.0)
Psychological	3 (1.1)	0 (0.0)	0 (0.0)	1 (1.3)	0 (0.0)	2 (4.3)

HPP-related manifestations and events considered to contribute to the clinical burden of HPP were identified by four clinical experts who had experience in treating patients with HPP. Bold italicized numbers indicate the three most frequent symptoms for each age group. Only patients who had been followed longitudinally for at least 1 year were included in this analysis

^a^Multiple fractures defined as three or more. ^b^Dental other includes delayed eruption of teeth, abnormality of the dentition, severe periodontitis, and atrophy of alveolar ridges. ^c^Only patients who survived at least 1 year were included in this analysis

*HPP* hypophosphatasia

### Age at occurrence of HPP-related manifestations or events considered to contribute to the clinical burden of HPP

**Fig. 2:**
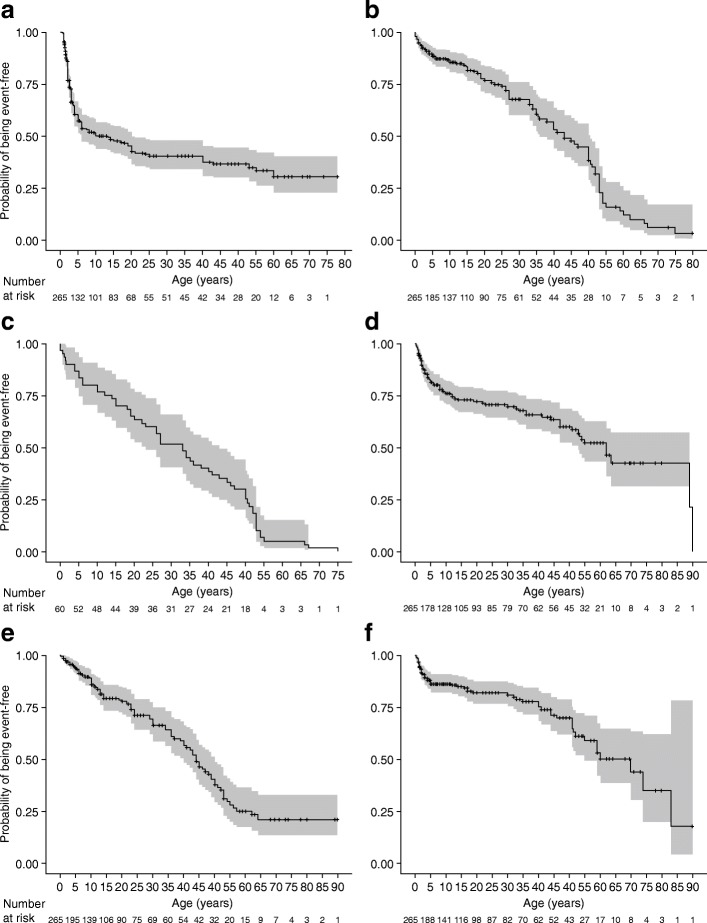
Kaplan–Meier curves for time to (**a**) premature tooth loss, (**b**) fracture^a^, (**c**) multiple fracture^b^, (**d**) gross motor/ambulation difficulties, (**e**) pain, and (**f**) surgery. Solid lines show the Kaplan–Meier estimation; grey shading denotes probability of being event-free between upper and lower 95% CI. Only patients who had been followed longitudinally for at least 1 year were included in this analysis. Patients were censored at first occurrence of the clinical symptom of interest, death, or loss to follow-up. ^a^Time to first fracture among patients with one or more fractures. ^b^Time to first fracture among patients with multiple fractures, defined as three or more. CI, confidence interval

**Fig. 3:**
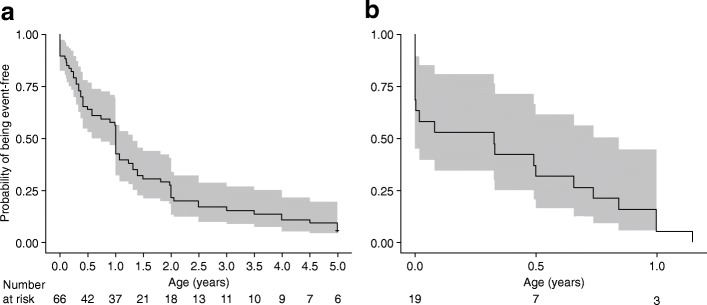
Kaplan–Meier curves for time to (**a**) cranial abnormalities and (**b**) respiratory symptoms in patients with these manifestations or events. Solid lines show the Kaplan–Meier estimation; grey shading denotes probability of being event-free between upper and lower 95% CI. Owing to the distribution of the data, only patients who had been followed longitudinally for at least 1 year and had experienced cranial abnormalities and/or respiratory systems before the age of 5 years were included in this analysis. The x axes in panels a and b are restricted to 5 years and 1 year, respectively. Patients were censored at first occurrence of the clinical symptom of interest, death, or loss to follow-up. CI, confidence interval

The median age at first occurrence of cranial abnormalities, pain, respiratory symptoms, premature tooth loss, fracture, gross motor/ambulation difficulties, and surgery were evaluated. Respiratory symptoms, cranial abnormalities, and premature tooth loss were first reported to occur in infancy and childhood (median age of occurrence ranged from 0.3 to 10 years), whereas fractures (one or more, or multiple [three or more]), pain, gross motor/ambulation difficulties, and surgery occurred in adulthood (median age of occurrence ranged from 33 to 70 years; Table 4). The median age at first occurrence of HPP-related manifestations or events, and the possible evolution of symptomology were found to vary when these data were evaluated by age at first HPP manifestation (Table 5). In addition, K–M analyses suggested that the probability of experiencing one of these manifestations or events increased with age (Figs. 2 and 3). Cranial abnormalities and respiratory symptoms, which are considered to be the most severe manifestations of HPP, were more likely to occur below the age of 1 year (only patients aged less than 5 years were included in this analysis; Fig. 3). Premature loss of teeth was common in childhood, but also occurred throughout adolescence and adulthood (i.e. the loss of permanent teeth). In pediatric patients with HPP, the median (95% confidence interval [CI]) age at first occurrence of premature loss of teeth was 3 (2–3) years; first occurrence of HPP-related surgery, fracture, pain, and gross motor/ambulation difficulties was between 2 and 13 years (data not shown). Among pediatric patients with HPP who were followed through adulthood, the risk of fracture, gross motor/ambulation difficulties, pain, and surgery in adulthood was high.

**Table 4:** Median age at first reported occurrence of manifestations/events considered to contribute to HPP clinical burden

Manifestation/event	Median age, years (range)
Respiratory symptoms	0.3 (0–1)
Cranial abnormalities	1 (0–12)
Premature loss of teeth	10 (0.5–60)
One or more fractures	43 (0–75)
Multiple fractures^a^	33 (0–75)
Pain	44 (0–64)
Gross motor/ambulation difficulties	62 (0.2–90)
Surgery	70 (0.1–83)

HPP-related manifestations/events considered to contribute to the clinical burden of HPP were identified by four clinical experts who had experience in treating patients with HPP. The data shown are the median age at first reported occurrence of such manifestations/events across all ages of first HPP manifestation. Only patients who had been followed longitudinally for at least 1 year were included in this analysis

^a^Multiple fractures defined as three or more

HPP, hypophosphatasia

**Table 5:** Median age at first reported occurrence of manifestations/events considered to contribute to HPP clinical burden, by age at occurrence of first HPP manifestation

Manifestation/event	Median age at first manifestation of HPP, years (range)				
	In utero *n* = 30	Infancy (0 to < 2 years) *n* = 102	Childhood (2 to < 10 years) *n* = 78	Adolescence (10 to < 18 years) *n* = 9	Adulthood (≥ 18 years) *n* = 46
Respiratory symptoms	0.1 (0–0.8)	0.4 (0–1)	N/A	N/A	N/A
Cranial abnormalities	1 (0–4)	0.7 (0–12)	5 (1–9)	N/A	N/A
Pain	1 (1–1)	34 (0–50)	40 (3–56)	42 (10–57)	51 (20–64)
Surgery	2 (0–3)	2 (0.1–30)	59 (1–59)	44 (12–44)	70 (32–83)
Gross motor/ambulation difficulties	2 (0.5–5)	22 (0–54)	18 (1–55)	19 (19–19)	89 (30–90)
Premature loss of teeth	3 (1–4)	3 (0–24)	5 (2–21)	14 (13–17)	25 (6–60)
One or more fractures	15 (0–15)	38 (0–53)	35 (2–55)	53 (15–53)	50 (19–75)

HPP-related manifestations/events considered to contribute to the clinical burden of HPP were identified by four clinical experts who had experience in treating patients with HPP. Only patients who had been followed longitudinally for at least 1 year were included in this analysis

*HPP* hypophosphatasia

## Discussion

This is the first study to report a comprehensive review of published HPP cases to characterize the clinical manifestations that are likely to contribute to the burden of HPP over time. Almost 95% of patients with HPP, regardless of when the disease first manifested, developed at least one such clinical manifestation or event during their follow-up. Premature loss of teeth, fracture, and pain were the most common manifestations. These results are in line with previous research into the clinical signs and symptoms associated with HPP, which found that pain [11, 12], dental abnormalities [13], and low-trauma fractures [7, 11, 14] were characteristic of the disease.

Several manifestations and events, such as fractures (one or more, or multiple), pain, gross motor/ambulatory difficulties, and surgery were reported more frequently with increasing age; however, the highest risk of cranial abnormalities and respiratory symptoms was observed for the youngest patients. These findings are in agreement with those of previous studies in the literature that show that severe respiratory impairment and cranial abnormalities are characteristic of HPP in young patients [2, 19].

Despite the widespread use of the classification of HPP based on age at first disease manifestation, it is still unclear whether these phenotypes reflect separate disease forms or are different stages of a progressive disease, particularly with respect to odonto-HPP. Our finding that multiple manifestations and events can accumulate over time and that the types of manifestations and events patients’ experience can change, particularly the evolution from dental abnormalities to skeletal manifestations later in life, supports the notion that HPP could be a progressive disease. It also highlights the importance of monitoring patients with HPP-related manifestations closely to identify worsening of their disease. Moreover, in one case series, eight out of nine patients who were diagnosed with childhood or adult-onset HPP and who experienced a substantial disease burden had initially presented with only dental manifestations of HPP (premature loss of primary teeth) as children [15]. This finding could support that odonto-HPP may be an early manifestation of HPP rather than a discrete disease entity, as previously suggested [15]. Prospective long-term observational or clinical studies would be required to assess disease burden and evolution rigorously. Importantly, improved understanding of disease burden and evolution may assist clinicians in identifying the patients with HPP who are most likely to benefit from enzyme replacement therapy [10].

The rarity of HPP and the limited availability of data restrict our understanding of the natural history and burden of this disease. The findings from this systematic literature review and synthesis of case reports highlight the novel approaches required to collate data on rare diseases where, because of small patient populations, few large-scale, rigorous observational or clinical studies can be conducted. Synthesizing case reports allows for the efficient use of available data, ensuring findings of all published case reports feed into our current understanding of the natural history and burden of a disease.

However, the use of case report data has inherent limitations, including the fact that clinical data are not routinely collected at regular follow-up visits as they would be in a prospective study. This lack of routinely collected follow-up data is important to consider when interpreting the findings of this study, particularly with regard to the impact of early censoring on the precise estimation of age at first occurrence of manifestations and events that contribute to the clinical burden of HPP. This is particularly important for those manifestations and events occurring later in life, at which time it is likely that a greater number of patients would have already been censored. In addition, as a result of publication bias, reports of patients with mild symptoms or with symptomology not deemed sufficiently important to describe may be less likely to be included in the scientific literature than reports of patients experiencing more pronounced symptomatology, possibly overestimating the prevalence of more severe manifestations. On the other hand, the exclusion of patients who did not survive beyond 1 year of life likely reduced the actual proportion of patients who were recorded as having been hospitalized or having respiratory symptoms, underestimating the prevalence of these severe manifestations/events. Despite these data limitations, we believe that the analyses undertaken provide valuable insight into the frequency and timing of manifestations that contribute to the clinical burden of HPP, given that the generation of more robust longitudinal data in orphan conditions like HPP is a major challenge.

## Conclusion

This approach using longitudinal, patient-level data collected from case reports is the first of its kind in HPP to synthesize those data to attempt to build on the current understanding of the natural history of the disease. Our findings confirm the high clinical burden of disease among patients with HPP, regardless of when the disease first manifests. In addition, we showed that the types of HPP-related clinical manifestations and events that individuals’ experience can change over time and multiple manifestations and events can accumulate over a lifetime. These observations may reflect evolution and progression of the disease; however, a comprehensive patient chart review, or similar, is needed to confirm the insights provided by the present study. The evidence gaps identified from this literature review can help to inform future research priorities that prospectively aim to characterize HPP-related symptoms that have a potential impact on disease burden.

## Additional files

Additional file 1: HPP-related clinical manifestation and event categories. (DOCX 15 kb)

Additional file 2: PRISMA flow diagram presenting the selection of eligible studies. PRISMA, Preferred Reporting Items for Systematic Reviews and Meta-Analyses. (EPS 1824 kb)

Additional file 3: Schematic illustrating the cohorts of patients with HPP included in the different analyses. HPP, hypophosphatasia. (EPS 1798 kb)

Additional file 4: Timing of HPP-related clinical manifestations/events in patients showing first manifestations in (a) *utero*, (b) adolescence, and (c) adulthood. **a** In utero (*n* = 30). **b** Adolescence; 10 to < 18 years (*n* = 9). **c** Adulthood; **i** 18–39 years (*n* = 22) and **ii** ≥ 40 years (*n* = 21). Cases are ordered by available follow-up. Only patients who had been followed longitudinally for at least 1 year and who experienced one or more HPP-related manifestations or events of interest were included in this analysis. ‘Resolution of symptoms’ was reported by the author of the case report. ‘Not reported’ defined as either nothing reported for this time period or no manifestations of interest. Anchor visit defined as the first point of contact with the author of the case report. HPP, hypophosphatasia; LTF, lost to follow-up. (EPS 2093 kb)

## Abbreviations

CI: Confidence interval
HPP: Hypophosphatasia
IQR: Interquartile range
K–M: Kaplan–Meier
odonto-HPP: Odontohypophosphatasia
PRISMA: Preferred Reporting Items for Systematic Reviews and Meta-analyses
