# Supplementary material for: Frequency and age at occurrence of clinical manifestations of disease in patients with hypophosphatasia: a systematic literature review
Source: Orphanet J Rare Dis. 2019 Apr 25;14:85. doi: 10.1186/s13023-019-1062-0 (PMC6485115; doi:10.1186/s13023-019-1062-0)
Supplement: Supplementary file 1 — HPP-related clinical manifestation and event categories. (DOCX 15 kb) [file 13023_2019_1062_MOESM1_ESM.docx]

**Additional file 1. HPP-related clinical manifestation and event categories**

| Manifestation/event category | Description |
| --- | --- |
| **Skeletal** | - Abnormal bone metabolism (osteomalacia, osteopenia, osteoporosis, hypomineralization, delayed ossification) |
|  | - Rickets-like bone deformities (abnormality of the metaphysis, genu valgum) |
|  | - Short long bone and/or bowing of the long bones |
|  | - Cranial abnormalities (craniosynostosis, frontal bossing, large anterior fontanel, decreased skull ossification, increased intracranial pressure, hydrocephalus, non-vitamin B6 responsive seizures) |
|  | - Abnormality of the ribs |
|  | - Fractures including pseudofracture and stress fracture |
|  | - Arthropathy including CPPD arthritis, pseudogout, arthritis, and chondrocalcinosis |
|  | - Pain (bone, joint, muscle, and nonspecific/generalized) |
|  | - Short stature or loss of height |
|  | - Scoliosis and/or kyphosis |
|  | - Bowdler spurs or dimples |
|  | - Talipes equinovarus |
| **Gross motor/ambulatory difficulties** | Delayed or missed gross motor milestones |
| **Dental** | - Premature loss of primary and/or permanent teeth |
|  | - Other dental symptoms (delayed eruption of teeth, abnormality of the dentition, severe periodontitis, and atrophy of alveolar ridges) |
| **Respiratory** | Respiratory symptoms (respiratory compromise, pulmonary hypoplasia, respiratory failure, recurrent respiratory tract infections, and need for respiratory support) |
| **Renal** | - Renal insufficiency |
|  | - Nephrocalcinosis |

CPPD, crystal pyrophosphate dihydrate; HPP, hypophosphatasia.
